# A retrospective analysis of the prognosis of prostate cancer patients with lymph node involvement on MR lymphography: who might be cured

**DOI:** 10.1186/1748-717X-8-190

**Published:** 2013-07-30

**Authors:** Hanneke JM Meijer, Oscar A Debats, Emile NJTh van Lin, Johannes Alfred Witjes, Johannes HAM Kaanders, Jelle O Barentsz

**Affiliations:** 1 Department of Radiation Oncology, Radboud University Nijmegen Medical Centre, P.O. Box 9101, 6500 HB Nijmegen, The Netherlands; 2 Department of Radiology, Radboud University Nijmegen Medical Centre, P.O. Box 9101, 6500 HB Nijmegen, The Netherlands; 3 Department of Urology, Radboud University Nijmegen Medical Centre, P.O. Box 9101, 6500 HB Nijmegen, The Netherlands

**Keywords:** Prostate cancer, Lymphotrophic superparamagnetic nanoparticles, Magnetic resonance imaging, Lymph node metastases, Radiotherapy

## Abstract

**Background:**

The prognosis of prostate cancer patients with lymph node metastases so small they can only be visualized by new imaging techniques as MR lymphography (MRL) is unknown. The purpose of this study was to investigate the prognosis of prostate cancer patients with non-enlarged metastatic lymph nodes on MRL and to identify a subgroup of MRL-positive patients who might be candidates for curative treatment.

**Methods:**

The charts of 138 prostate cancer patients without enlarged lymph nodes on CT, in whom a pre-treatment MRL was performed were reviewed. Endpoints were distant metastases-free survival and overall survival. Relation between the following factors and outcome were investigated: T-stage, PSA value at diagnosis, Gleason score, diameter (short axis and long axis) of the largest MRL-positive lymph node, number of MRL-positive lymph nodes, the presence of extra-pelvic nodal disease, and the extent of resection of the positive lymph nodes. Kaplan-Meier analysis was performed to estimate the survival functions.

**Results:**

Of the 138 patients, 24 (17%) had a positive MRL. Patients with a short axis of the largest positive lymph node of ≤8 mm had a significantly better 5-year distant metastases-free (79% vs 16%) and overall survival (81% vs 36%) than patients with larger positive lymph nodes. This also accounted for patients with a largest long axis of ≤10 mm (71% vs 20% and 73% vs 40%, respectively). Outcome was also better in patients in whom all positive lymph nodes had been resected.

**Conclusion:**

A selection of MRL-positive patients with a good prognosis could be identified, consisting of patients with small positive lymph nodes. In these patients, cure might be pursued.

## Background

Lymph node metastases in prostate cancer patients can only be detected with conventional imaging methods as computed tomography (CT) or magnetic resonance imaging (MRI) when nodal enlargement exists, which does not occur until late in the disease process [1]. Nodal involvement is therefore generally detected through a lymph node dissection. Patients with nodal involvement are historically considered to be incurable [2]. However, several studies have shown that their prognosis is highly variable [3,4]. Patients with a low nodal metastastic tumor burden have a favorable outcome after prostatectomy and lymph node dissection. They can remain disease-free for many years [2].

The development of new imaging methods, such as MR lymphography (MRL), has created the opportunity to detect lymph node metastases at an early stage, in non-enlarged lymph nodes [5]. MRL is a technique that uses the contrast agent ferumoxtran-10 to enhance MRI. This method has a sensitivity of 80-100% and a specificity of 87-99% for the detection of involved lymph nodes in prostate cancer [6,7].

Patients in whom lymph node metastases are diagnosed non-invasively at an early stage, before enlargement of the lymph node occurs, form a new category, with an unknown prognosis. Their outcome might be comparable to that of patients with clinically occult metastases detected by pelvic lymph node dissection.

With this new category of node-positive patients, a treatment dilemma has arisen. Should we pursue cure with surgery or locoregional radiotherapy, and if yes, how should we select those patients that are most likely to benefit? To address this issue, it is necessary to obtain more data are about the course of the disease in these patients.

The purpose of the present study was to investigate the prognosis of CT-negative patients with positive lymph nodes on MRL and to identify a subgroup of MRL positive patients with a good prognosis, who might be candidates for curative treatment.

## Methods

### Patient selection

Between January 2003 and May 2005, 150 patients with a histopathologically proven intermediate to high-risk prostate cancer (serum prostate-specific antigen level >10 ng/mL, Gleason score >6, or T3 clinical stage), with lymph nodes with a maximum diameter of 1.5 cm on CT, underwent an MRL prior to local treatment in our institute in the context of a pathological validation study. In all patients, histopathological evidence was obtained, either by pelvic lymph node dissection that was in some cases extended based on MRL, or by CT-guided lymph node biopsy. Twelve patients were excluded for the analysis as follow-up data were not available or incomplete. The charts of the remaining 138 patients were reviewed.

The study was approved by the Institutional Review Board: Commissie Mensgebonden Onderzoek regio Arnhem-Nijmegen and written informed consent was obtained from all patients.

### MRL scanning procedure

MRI images were obtained on a 1.5 T system (Sonata/Symphony, Siemens, Erlangen, Germany; Gyroscan/Intera, Philips, Eindhoven, Netherlands; or Horizon, GE Medical Systems, Milwaukee, WI, USA) with pelvic phased array coils. To suppress bowel peristalsis, Buscopan i.m. and i.v., and Glucagon i.m. were administered before scanning. Patients were placed in the supine position with a knee fix. Images were acquired from the entire pelvis and abdomen. Heesakkers *et al.* previously described the scanning protocol in detail [6].

Twenty-four to 36 hours before MRI, Ferumoxtran-10 (Sinerem®, Guerbet, Paris, France) was injected intravenously. This contrast medium contains ultrasmall superparamagnetic particles of iron oxide. These particles extravasate, and are transported to the lymph nodes by macrophages. Iron particles give a low signal intensity on a T2*-weighted MRI image. Metastases in the lymph nodes block accumulation of the iron particles. The signal intensity of pathological nodes will therefore remain high on a T2*-weighted MRI image, while the signal intensity of normal lymph nodes becomes low [6,8].

Lymph nodes were considered malignant when they completely or partially showed high signal intensity on a T2*-weighted image [6]. All MRL images were analyzed by an experienced radiologist.

### Treatment and follow-up after MRL

After MRL, patients were treated for their disease, according to the result of the histopathological examination. MRL patients that were histopathologically node-negative were locally treated with prostatectomy or local radiotherapy, patients that were node-positive received hormonal treatment. Two node-positive patients did undergo a prostatectomy, because lymph node dissection and prostatectomy were combined in one procedure after MRL was negative. Follow-up took place every 3–6 months, for a minimum of 5 years. At every visit, a PSA value was determined. A CT, MRI or bone scan was performed at the physician’s discretion. In case of a recurrence patients were treated depending on the site of recurrence.

### Endpoints and statistical analysis

For statistical testing SPSS 16.0.01 (SPSS Inc. 1989–2007) was used and a p < 0.05 was a priori deemed significant.

Endpoints of the present study, that were determined for patients with and without lymph node involvement on MRL, were: distant metastases-free survival (DMFS) and overall survival (OS). For DMFS, deaths were censored. Survival rates were estimated with the Kaplan-Meier method, and the unstratified log-rank statistical analysis was used to test for differences.

### Patients with a positive MRL

For patients with a positive MRL, the prognostic value of tumor-related factors was investigated using Kaplan-Meier analysis: T-stage, Gleason score (<7, 7 or >7), PSA (≤10 ng/ml, >10 ng/ml).

Next, MRL-related factors were investigated: number of positive lymph nodes, largest diameter of the largest lymph node (mm), short axis diameter of the largest lymph node (mm).

For these factors, ROC analysis was performed to determine their predictive potential and to find the threshold with the highest accuracy. This threshold was used to dichotomize the patients for Kaplan-Meier analysis.

Further, the prognostic value of the presence or absence of positive lymph nodes outside the pelvis was determined. Positive lymph nodes above the L5/S1 interspace were considered to be outside the pelvis.

Last, it was investigated whether patients in whom all MRL-positive lymph nodes had been removed had a better prognosis compared to patients in whom only part of the MRL-positive lymph nodes had been removed. To determine whether all MRL-positive lymph nodes had been removed, a comparison between the surgical report, the pathology report, and the MRL result was made.

Multivariate analysis could not be performed, due to the relatively small number of MRL-positive patients.

## Results

### Total study population

**Figure 1:**
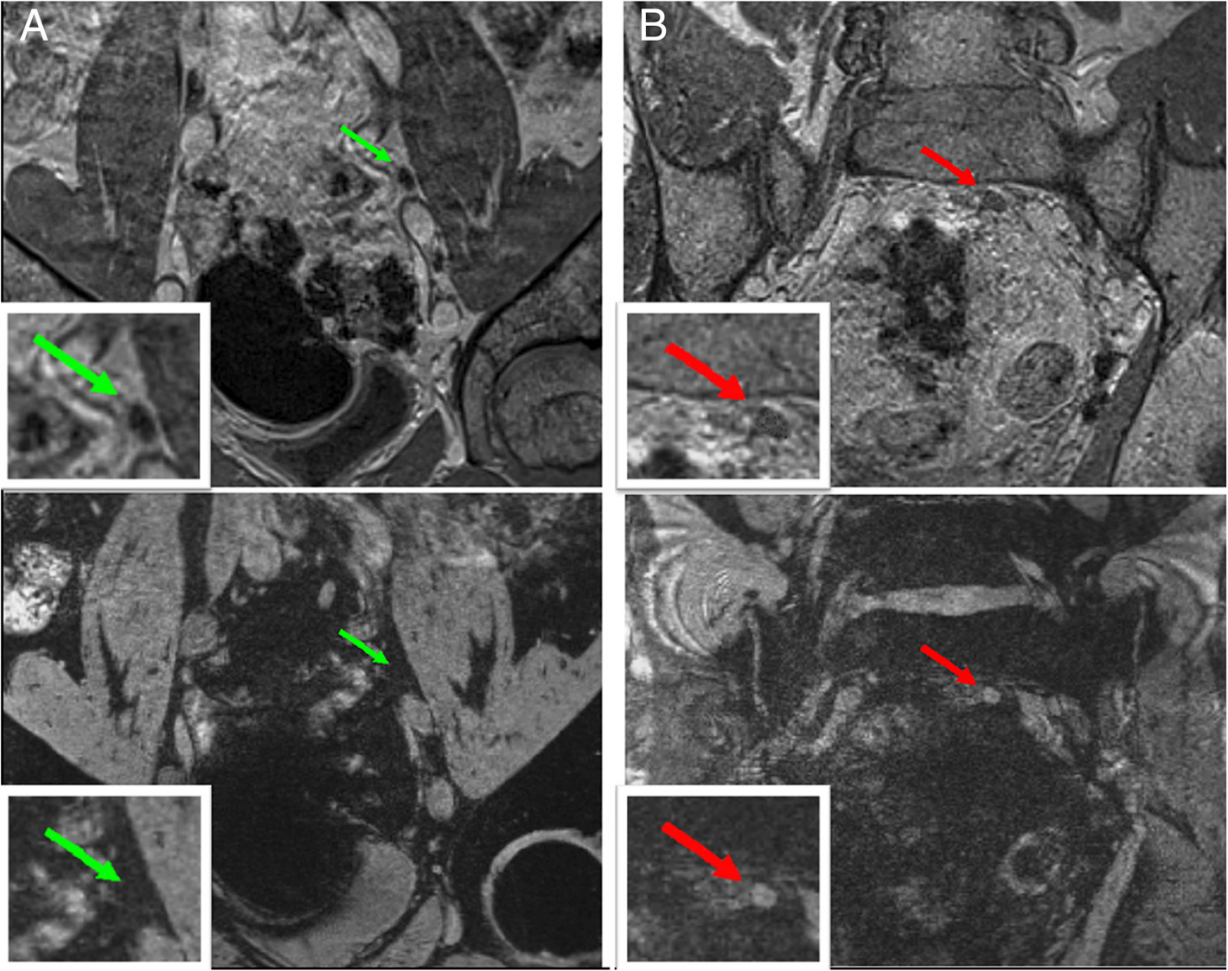
***Example of a negative (A) and a positive (B) MRL. ****Upper panel*: T1 image, which provides good visualization of non-enlarged lymph nodes (arrows). *Lower panel:* T2* image. The positive lymph node **(B)** has a high signal intensity, because accumulation of the iron particles has been blocked. This in contrast to the low signal intensity of the negative lymph node **(A)**. *Modified from: Hanneke J.M. Meijer et al.; Magnetic resonance lymphography findings in patients with a biochemical recurrence after prostatectomy and the relation with the Stephenson nomogram. Int. J. Radiat. Oncol. Biol. Phys. 2012; 84(5): 1186–1191.*

The characteristics of the 138 patients are shown in Table 1. Twenty-four patients had a positive MRL (17%). Figure 1 shows an example of an MRL-positive and an MRL-negative lymph node. In 2 patients with a negative MRL, lymph node involvement was found at histopathological verification. In 10 patients with a positive MRL, histopathological examination was negative.

**Table 1:** Patient characteristics

		**Median**
		**(range)**
PSA		15.9 (2.8-260.0)
		N (%)
Clinical T-stage	1	31 (22%)
	2	85 (62%)
	3	22 (16%)
Gleason score	Unknown	1 (1%)
	5	4 (3%)
	6	32 (23%)
	7	62 (45%)
	8	26 (19%)
	9	10 (7%)
	10	3 (2%)
MRL Result	Negative	114 (83%)
	Positive	24 (17%)
Result PA	Negative	122 (88%)
	Positive	16 (12%)
Therapy	Prostatectomy	77 (56%)
	Radiotherapy with neo-adjuvant hormonal treatment	47 (34%)
	Hormonal therapy	14 (10%)

*Abbreviations: MRL* magnetic resonance lymphography, *PA* pathology.

**Figure 2:**
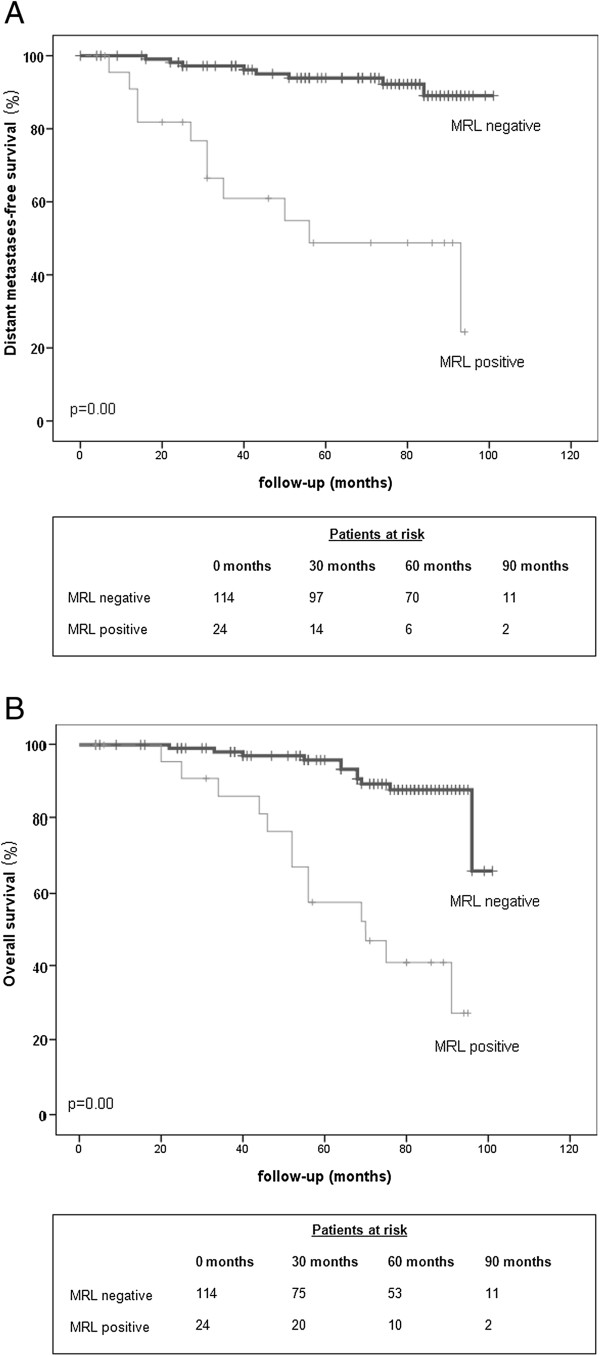
***Survival curves for MRL negative vs MRL positive patients. *A**. Distant metastases-free survival. **B**. Overall survival. p-value indicates level of statistical significance as determined with unstratified log-rank statistical analysis Max = maximum.

Median follow-up time for all patients was 73 months (range 4–101 months). Figure 2 shows the Kaplan-Meier survival curves for DMFS (A) and OS (B) for patients with a positive and for patients with a negative MRL. Five-year DMFS was 94% for the MRL negative group, and 49% for the MRL positive group. Five-year OS was 96% and 57% respectively.

### Patients with a positive MRL

The Kaplan-Meier survival curves for both DMFS and OS did not differ significantly between patients with a PSA ≤10 ng/ml and >10 ng/ml, different clinical T-stages, or different Gleason scores (<7, 7 or >7).

In ROC analysis, the number of positive lymph nodes was not predictive for either endpoint. The short axis of the largest positive lymph node was predictive for the occurrence of distant metastases (area under the curve (AUC) 0.76 with p = 0.03). The threshold at which the highest accuracy (75%) was reached was 8 mm. The short and the long axis of the largest lymph node were both predictive of OS (AUC 0.82 with p = 0.01; AUC 0.74 with p = 0.05 respectively). Thresholds with the best accuracy were 8 mm for the short axis and 10 mm for the long axis diameter of the largest lymph node (accuracy 84% and 76% respectively).

Based on these thresholds, patients were dichotomized. Of the group with a short axis of the largest lymph node of ≤8 mm, only 1 patient had a long axis of the largest lymph node of >10 mm. Vice versa, of the patients with a long axis of the largest lymph node of ≤10 mm, also only 1 patient had a lymph node with short axis of >8 mm.

**Figure 3:**
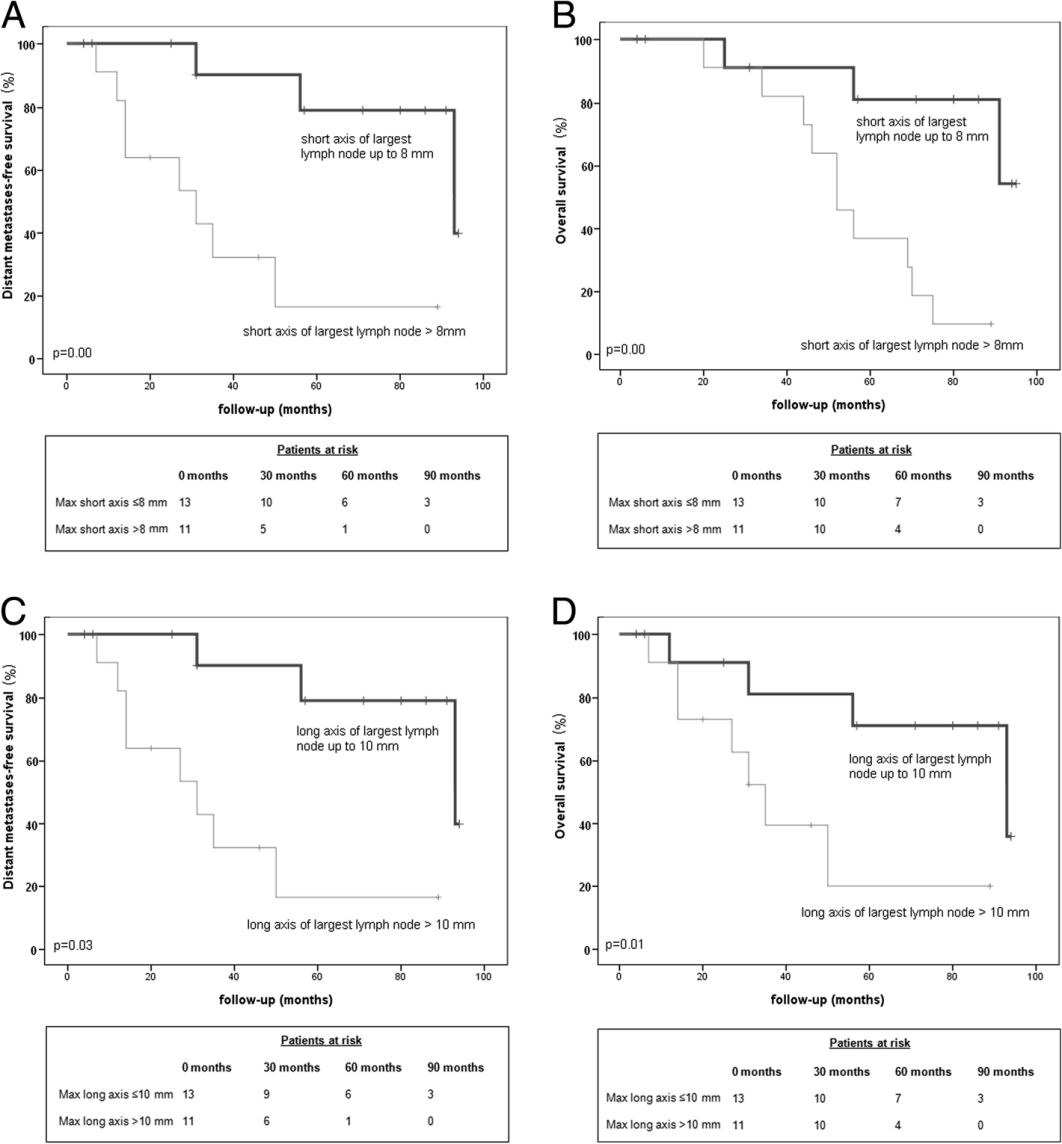
***Survival curves for subgroups of the MRL positive patients with different MRL related factors. ****Upper panel*: Patients with a short axis of the largest lymph node of ≤8 mm vs patients with a short axis of the largest lymph node of >8 mm. **A**. Distant metastases-free survival. **B**. Overall survival. *Lower panel*: Patients with a long axis of the largest lymph node of ≤10 mm vs patients with a long axis of the largest lymph node of >10 mm. **C**. Distant metastases-free survival. **D**. Overall survival. p-value indicates level of statistical significance as determined with unstratified log-rank statistical analysis Max = maximum.

Figure 3 shows the corresponding survival curves. DMFS and OS were significantly better for patients in whom the short axis of the largest positive lymph node was ≤8 mm. Five-year DMFS was 79% versus 16% for the group with a longer short axis. Five-year OS was 81% and 36%, respectively. Similar results were obtained after dichotomization by the long axis size. Five-year DMFS was 71% and 20% respectively, 5-year OS 73% and 40% respectively.

There were 7 patients with extra-pelvic nodal disease. Their distant metastases-free and overall survival did not significantly differ from those of patients with nodal disease limited to the pelvis.

In eight patients all MRL positive lymph nodes had been removed. These patients had a significantly better 5-year DMFS (80%) than patients in whom only part of the positive lymph nodes had been removed (35%; p = 0.04). OS did not differ between the groups.

## Discussion

The presence of lymph node metastases in prostate cancer is a poor prognostic sign [2], and patients with lymph node metastases generally are considered incurable [2,4]. The present study shows that patients with a negative MRL indeed have a far better outcome than MRL-positive patients. However, within the group of MRL-positive patients, a subgroup could be defined with a better prognosis, consisting of patients with only small nodal metastases. In these patients cure might be pursued, for example by locoregional radiotherapy or resection of the positive nodes.

CT and MRI have a very low sensitivity for the detection of lymph node metastases in prostate cancer [9]. The presence of lymph node metastases is therefore usually detected at pelvic lymph node dissection. However, the development of more accurate imaging methods has created the opportunity to detect lymph node involvement at an early stage, in a non-invasive manner. MRL is a very accurate imaging method, with a sensitivity of 85-100%, which can detect lymph node metastases even in non-enlarged lymph nodes [6,7].

Thus a new category of patients emerges, whose prognosis and the treatment from which they benefit require further investigation. The present study aimed to take the first steps in describing this group of patients.

MRL-positive patients had a significantly worse prognosis compared to MRL-negative patients. Five-year OS was 57%, which is comparable to the survival of lymph node positive patients treated with androgen suppression therapy alone in other reports [10]. However, patients with small MRL positive lymph nodes (a short axis of the largest positive lymph node of ≤8 mm or a long axis of ≤10 mm), had a 5-year DMFS and OS of more than 70%. This was despite the fact that these patients were treated palliatively. This is comparable to the prognosis of high-risk node-negative patients treated with radiotherapy [11,12]. Patients in whom all positive lymph nodes had been removed had the best prognosis.

These findings reinforce the results from previous studies investigating outcome after prostatectomy and lymph node dissection. These retrospective studies have shown that patients with limited lymph node involvement have a far better outcome compared to patients with more extensive lymph node involvement [2,4]. The number of positive lymph nodes [3,4] and the size of the largest positive lymph node [2] were prognostic factors. Disease-specific survival was 99% at 5 years for patients with a single lymph node metastasis [4], 84% at 15 years for patients with up to 2 positive lymph nodes [3], and over 80% at 5 years for patients in whom the largest positive lymph node was ≤ 10 mm. Five-year recurrence-free survival of the latter group was around 40% [2].

Although patients with limited lymph node involvement seem to have a better outcome compared to patients with more extensive nodal disease, the question remains: can they be cured? The answer may be ‘yes’ for at least part of the MRL-positive patients. The finding of the present study that DMFS is high for patients with limited nodal involvement, indicate a window of opportunity for cure with locoregional treatment, before distant metastases develop. This was also found by Leibel et al., who described the outcome in patients with nodal disease treated with pelvic lymphadenectomy and brachytherapy [13]. N1 (single lymph node metastasis <2 cm) patients had a better DMFS than N2 (multiple lymph node metastases or single lymph node metastasis >2 cm but <5 cm) patients. This is further supported by the results of the above mentioned studies [2-4]. Our finding that patients in whom all MRL-positive lymph nodes had been removed at pelvic lymph node dissection had a very high 5-year DMFS of 80%, further substantiate this. This is also in line with the findings of several retrospective studies indicating that the larger the extent of a lymph node dissection, and thus the higher the chance of removal of all nodal disease, the better the prognosis [14,15].

Preferably, only lymph node positive patients without distant micrometastases would be selected for locoregional treatment. The findings of the present study that patients with small MRL-positive lymph nodes less often and less early develop clinically apparent distant metastases, can guide MRL-based patient selection for a potentially curative locoregional treatment. When selecting node positive patients for locoregional treatment with MRL, the size of the short axis of the largest lymph node should be the main selection criterion, as there was significant overlap between the group with a short axis of the largest MRL-positive lymph node of ≤8 mm and the group with a long axis of ≤10 mm, with the short axis being a stronger prognostic factor at ROC analysis.

MRL can yield important progress in locoregional treatment for node positive patients. First, geographical miss of positive lymph nodes can be reduced. Studies using the sentinel node procedure or MRL to map the pattern of lymph drainage in prostate cancer patients, have shown that this is a larger problem than was previously thought. Positive lymph nodes and sentinel nodes were found outside the area of routine lymph node dissection [16], as well as outside the standard target volume for elective pelvis irradiation in more than half of the patients [17,18]. The possibility to target pathological lymph nodes more accurate, will increase the effectiveness of locoregional treatment in these patients.

Further, for radiotherapy, the use of MRL gives the opportunity to boost the positive lymph nodes. This has shown to be theoretically feasible, using an intensity modulated radiotherapy technique [19]. Dose escalation to the prostate has shown to improve outcome [20], and this may also account for the involved lymph nodes.

A limitation of the present study is the small number of MRL-positive patients. The findings of this study should therefore be interpreted as a first step towards identification of MRL-positive patients with a good prognosis in whom cure might be feasible.

A second limitation is that histopathological examination was negative in 10 of 24 MRL- positive patients. In 4 of these patients, however, the tissue sampling was not representative, because the MRL-positive lymph nodes were not removed, as was shown by a repeated MRL within 3 months after the initial MRL. We elected to include all MRL-positive patients for analysis. This because the aim of the present study was to find prognostic factors based on MRL without subsequent histopathological confirmation. This might have influenced our results, certainly as the histopathologically negative patients received a different (curative) treatment. As the group was too small for multivariate analysis, the influence of this factor on outcome relative to that of the prognostic factors found in our study could unfortunately not be investigated.

## Conclusions

Whereas lymph node involvement on MRL was a poor prognostic factor, a subgroup of MRL-positive patients with a relatively good prognosis could be identified. Patients with a short axis of the largest lymph node ≤8 mm or a long axis ≤10 mm had a good outcome, comparable to that of high-risk node-negative prostate cancer patients. These seem to be the patients in whom cure might be pursued. Patients in whom all MRL-positive lymph nodes had been resected, had a better prognosis than patients in whom part of the nodal disease was left in situ. This encourages the application of a locoregional curative treatment in these patients.

## Abbreviations

MRL: Magnetic resonance lymphography; DMFS: Distant metastases-free survival; OS: Overall survival; ROC: Receiver operating characteristic; PA: Pathology; Max: maximum.

## Competing interests

The authors declare that they have no competing interests.

## Authors’ contributions

HM reviewed the charts of the patients’ en analysed the data. Further, she wrote the manuscript. OD, EL, JW, JK and JB contributed to the conception and design of the study, and revised the manuscript critically. All have given final approval of the version to be published.
